# Distribution and efficacy of ofatumumab and ocrelizumab in humanized CD20 mice following subcutaneous or intravenous administration

**DOI:** 10.3389/fimmu.2022.814064

**Published:** 2022-07-28

**Authors:** Julia Baguña Torres, Jay Roodselaar, Megan Sealey, Marina Ziehn, Marc Bigaud, Rainer Kneuer, David Leppert, Gisbert Weckbecker, Bart Cornelissen, Daniel C. Anthony

**Affiliations:** ^1^ Department of Oncology, University of Oxford, Oxford, United Kingdom; ^2^ Department of Pharmacology, University of Oxford, Oxford, United Kingdom; ^3^ Novartis Pharma AG, Basel, Switzerland; ^4^ Novartis Institutes for Biomedical Research, Basel, Switzerland; ^5^ Department of Neurology, University Hospital Basel, Basel, Switzerland

**Keywords:** ofatumumab, ocrelizumab, subcutaneous, intravenous, multiple sclerosis, distribution, B-cells

## Abstract

Approval of B-cell-depleting therapies signifies an important advance in the treatment of multiple sclerosis (MS). However, it is unclear whether the administration route of anti-CD20 monoclonal antibodies (mAbs) alters tissue distribution patterns and subsequent downstream effects. This study aimed to investigate the distribution and efficacy of radiolabeled ofatumumab and ocrelizumab in humanized-CD20 (huCD20) transgenic mice following subcutaneous (SC) and intravenous (IV) administration. For distribution analysis, huCD20 and wildtype mice (n = 5 per group) were imaged by single-photon emission computed tomography (SPECT)/CT 72 h after SC/IV administration of ofatumumab or SC/IV administration of ocrelizumab, radiolabeled with Indium-111 (^111^In-ofatumumab or ^111^In-ocrelizumab; 5 µg, 5 MBq). For efficacy analysis, huCD20 mice with focal delayed-type hypersensitivity lesions and associated tertiary lymphoid structures (DTH-TLS) were administered SC/IV ofatumumab or SC/IV ocrelizumab (7.5 mg/kg, n = 10 per group) on Days 63, 70 and 75 post lesion induction. Treatment impact on the number of CD19+ cells in select tissues and the evolution of DTH-TLS lesions in the brain were assessed. Uptake of an ^111^In-labelled anti-CD19 antibody in cervical and axillary lymph nodes was also assessed before and 18 days after treatment initiation as a measure of B-cell depletion. SPECT/CT image quantification revealed similar tissue distribution, albeit with large differences in blood signal, of ^111^In-ofatumumab and ^111^In-ocrelizumab following SC and IV administration; however, an increase in both mAbs was observed in the axillary and inguinal lymph nodes following SC versus IV administration. In the DTH-TLS model of MS, both treatments significantly reduced the ^111^In-anti-CD19 signal and number of CD19+ cells in select tissues, where no differences between the route of administration or mAb were observed. Both treatments significantly decreased the extent of glial activation, as well as the number of B- and T-cells in the lesion following SC and IV administration, although this was mostly achieved to a greater extent with ofatumumab versus ocrelizumab. These findings suggest that there may be more direct access to the lymph nodes through the lymphatic system with SC versus IV administration. Furthermore, preliminary findings suggest that ofatumumab may be more effective than ocrelizumab at controlling MS-like pathology in the brain.

## Introduction

Multiple sclerosis (MS) is a chronic autoimmune, demyelinating disease of the central nervous system (CNS), associated with a complex and varied array of sensory, motor and cognitive symptoms (1). Most patients with MS follow an initial relapsing-remitting disease course, characterized by episodes of disease activity with full or partial recovery of neurological function (2, 3). This is often followed by a secondary progressive course, in which gradual worsening of disability occurs over time, with no or few relapses (2–4).

Historically, MS has been considered a T-cell-mediated disorder; however, we now know that the cellular immunology of relapsing MS involves multiple cell types, including B-cells, and their functionally distinct subtypes (5). Beyond peripheral compartments (i.e. blood and secondary lymphoid organs), B-cells are found in distinct CNS compartments of patients with MS, including the cerebrospinal fluid (CSF) and parenchymal lesions (6, 7). Immune cell aggregates, rich in B-cells, have also been reported in the meninges of patients with both relapsing-remitting and secondary progressive disease (8–12). These meningeal B-cell aggregates, resembling tertiary lymphoid structures (TLS), are associated with more aggressive disease progression and with sites of underlying demyelination, microglial activation and neurodegeneration (8, 9, 11–15). Precisely how B-cells contribute to MS disease pathology remains unclear, though it is likely through a range of B-cell responses, including antigen presentation, autoantibody production, and cytokine secretion (16–19).

The most compelling proof for the contribution of B-cells to MS disease pathology comes from the success of B-cell-depleting anti-CD20 antibody therapies (20–23). CD20 is a transmembrane, non-glycosylated phosphoprotein expressed on the surface of pre-, immature, mature, and memory B-cells, but lost following differentiation into plasma cells (24). Rituximab, a genetically engineered chimeric monoclonal antibody (mAb), was the first anti-CD20 agent to demonstrate efficacy in relapsing MS (20, 21), and paved the way for the development of less immunogenic and possibly more potent humanized (ocrelizumab) and fully human (ofatumumab) anti-CD20 mAbs. In randomized phase 3 clinical trials, ocrelizumab (OPERA I and OPERA II) and ofatumumab (ASCLEPIOS I and ASCLEPIOS II) demonstrated superior efficacy to interferon beta-1a and teriflunomide, respectively, including robust reductions in annualized relapse rate and inflammatory disease activity (22, 23). Ocrelizumab was the first anti-CD20 mAb approved for use in MS, and is indicated for the treatment of relapsing and primary progressive forms of the disease (25, 26). Approval of ofatumumab for the treatment of relapsing MS was granted by the US Food and Drug Administration in August 2020 (27) and by the European Medicines Agency in March 2021 (28), with review and approval in other countries worldwide ongoing. Both anti-CD20 mAbs recognize distinct epitopes on CD20 (29) and have partly different modes of action, with ocrelizumab inducing B-cell depletion primarily *via* antibody-dependent cellular cytotoxicity (ADCC) and ofatumumab primarily *via* complement-dependent cytotoxicity (CDC) (30, 31). While mode of action may play a role in treatment outcome, it is hypothesized that the route of administration of these anti-CD20 mAbs could also have a profound impact (32).

The availability of transgenic mice expressing human CD20 (huCD20 mice) provides a means to understand the relative contribution of the route of administration and dose of these antibodies to the depletion of B-cells in the spleen, lymph nodes, and brain of mice. Furthermore, the recent development of a new mouse model of MS, which recapitulates the principle histopathologic features of the disease, including meningeal B-cell aggregates (33), enables better assessment of the efficacy of B-cell targeted therapies. Thus, the aim of this study was 1) to determine whether subcutaneous (SC) versus intravenous (IV) administration of ofatumumab or ocrelizumab alters antibody distribution in huCD20 mice; 2) to assess whether anti-CD20-mediated B-cell depletion can be detected with an indium (^111^In)-labelled anti-CD19 antibody; and 3) to establish how the administration route affects the efficacy of anti-CD20 mAbs in a huCD20 mouse model of MS.

## Materials and methods

### Mice

Adult huCD20 C57BL/6 mice (B6.FVB.Tg[CD20]Gne), expressing human CD20 exclusively on B-cells (34), and their wildtype (WT) littermates (JANVIER LABS, France) were housed in standard laboratory cages (3–5 per cage) in a controlled enriched environment with a 12:12 h light–dark cycle, and with food and water available *ad libitum*. All animal procedures were approved by the UK Home Office under license P996B4A4E and performed in accordance with the guidelines of the European Community Council Directives 2010/63/EU.

### Induction of DTH mouse model (pilot study)

Chronic delayed-type hypersensitivity (DTH) lesions, which no longer exhibit blood–brain barrier (BBB) breakdown, were established in huCD20 mice, as previously described (35). Briefly, mice were anaesthetized with 2–3% isoflurane in a mixture of nitrous oxide/oxygen (70%/30%) and placed in a stereotaxic frame. A midline incision was made in the scalp, and a burr hole was drilled 0.5 mm anterior and 1.5 mm lateral to bregma. Using a finely drawn glass microcapillary, 1 µL of saline containing 10^5^ organisms of heat-killed Bacillus Calmette–Guérin (BCG) (F. Hoffman-La Roche Ltd., Switzerland) was injected stereotactically into the left striatum. The lesion was activated 28 days later with a peripheral intradermal injection of BCG in complete Freund’s adjuvant into the hind limbs (Day 0). Mice were checked daily, and their wellbeing was ensured by monitoring their body weight and physical appearance.

Mice (n = 5 per group) were treated with SC or IV rituximab (7.5 mg/kg on Days 60 and 67 post lesion induction; F. Hoffman-La Roche Ltd., Switzerland), or vehicle. The impact of rituximab on T-cell accumulation, astrocyte reactivity and microglia activation in the DTH lesion was determined following 14 days of treatment.

### Induction of DTH-TLS mouse model

Focal MS-like lesions with TLS were established in huCD20 mice as previously described (33). Mice (N = 64) were first immunized with an emulsion of myelin oligodendrocyte glycoprotein (MOG)_35-55_ peptide (0.5 mg/mL in saline; Innovagen, Sweden) and complete Freund’s adjuvant with heat-killed Mycobacterium tuberculosis (TB) (Difco H37Ra, BD, UK; 5 mg/mL in the total volume), administered intradermally in the hind legs. Mice were anaesthetized 12 days later with 2–3% isoflurane in a mixture of nitrous oxide/oxygen (70%/30%) and placed in a stereotaxic frame. The head of the animal was shaved and sterilized. A midline incision was then made in the scalp, and a burr hole was drilled 0.7 mm anterior and 2.7 mm lateral to bregma. Using a finely drawn glass microcapillary, 1 µL of heat-killed TB (8.8 mg/mL in saline) was injected stereotactically into the piriform cortex (Day 0). Mice were checked daily, and their wellbeing was ensured by monitoring their body weight and physical appearance.

### ^111^In labeling of ofatumumab, ocrelizumab, and anti-CD19 antibody

Ofatumumab (0.3 mg; supplied by Novartis, Basel), ocrelizumab (0.3 mg; supplied by a pharmacy in Basel), and anti-mouse CD19 antibody (0.3 mg; produced in-house, **Supplementary Materials, Appendix A**) were each reacted with a 20-fold molar excess of p-SCN-Bn-DTPA in Chelex-100-treated 0.1 M sodium bicarbonate buffer (pH 8.6) for 2 h at 37°C. The complex was then purified on a 1 mL Sephadex G50 column, using 0.5 M MES buffer as the eluent. The DTPA-conjugated antibodies were then concentrated using an Amicon Ultra 0.5 mL 30 K MWCO filter unit.

DTPA-conjugated ofatumumab (DTPA-ofatumumab; 0.1 mg), DTPA-ocrelizumab (0.1 mg) and DTPA-anti-CD19 (0.1 mg) were each radiolabeled with ^111^In (0.5–1.0 MBq per mg of antibody) for 1 h at room temperature and purified in phosphate buffered saline (PBS; pH 7.4) using Zeba™ spin desalting columns (40K MWCO, Thermo Fisher Scientific, Massachusetts, USA). Radiolabeling efficiencies were determined by instant thin-layer chromatography (iTLC), and final antibody concentrations were measured by Nanodrop spectrophotometry (Thermo Fischer Scientific, Massachusetts, USA).

Antibody integrity was assessed by sodium-dodecyl sulfate polyacrylamide gel electrophoresis (SDS-PAGE). Briefly, DTPA-conjugated and ^111^In labelled antibodies were loaded (5 µg/well of each antibody) onto a 4–12% Bis-Tris gel in 1X MES and ran at 200 V for 30 min. The polyacrylamide gels were subsequently transferred to filter paper, wrapped in clingfilm, and storage phosphor autoradiography (Super Resolution, 12.5 x 12.5 cm; PerkinElmer, UK) was performed for 1 h to check the incorporation of radioactivity in the antibodies after radiolabeling.

### Distribution of ofatumumab and ocrelizumab using SPECT/CT

Both huCD20 and WT mice (n = 5 per group) were imaged by single-photon emission computed tomography (SPECT)/CT using VECTor^4^CT (MILabs, the Netherlands; see **Supplementary Materials** for further details, **Appendix A**) 72 h after SC/IV administration of ^111^In-ofatumumab or SC/IV administration of ^111^In-ocrelizumab (5 µg, 5 MBq) (reconstructed resolution, <1.2 mm; sensitivity, >4500 cps/MBq). The uptake of ^111^In per gram in the axillary and inguinal lymph nodes was quantified by volume-of-interest (VOI) image analysis and expressed as a percentage of injected dose per milliliter (%ID/mL).

Following SPECT/CT whole-body imaging, mice were euthanized and selected tissues were removed, immediately rinsed with water, blot dried, and weighted; blood was also collected. The amount of ^111^In in each sample was measured using a HiDex gamma counter (LabLogic Systems, UK). The percentage of the injected dose per gram (%ID/g) of each sample was calculated.

### B-cell imaging with ^111^In-anti-CD19

To assess whether ^111^In labeling of the CD19 epitope of B-cells could be used as an *in vivo* measure of B-cell depletion in tissues that have been targeted with the anti-CD20 mAbs, both huCD20 and WT mice (n = 5 per group) were imaged by SPECT/CT 72 h after SC or IV administration of ^111^In-anti-CD19 (5 µg, 5 MBq). The uptake of ^111^In per gram in the axillary and inguinal lymph nodes was quantified by VOI image analysis. Following SPECT/CT whole body imaging, mice were euthanized and their blood collected. Selected tissues were removed, immediately rinsed with water, blot dried, and transferred to pre-weighted counting tubes. The amount of ^111^In in each sample was measured as described above.

### Determination of ^111^In-anti-CD19 uptake following SC/IV administration of ofatumumab or SC/IV administration of ocrelizumab in the DTH-TLS mouse model

huCD20 mice with chronic DTH-TLS lesions (N = 16) were imaged by SPECT/CT 72 h after SC administration of ^111^In-anti-CD19 (5 µg, 5 MBq; 60 days post lesion induction) in order to assess B-cell numbers using the CD19 epitope. Mice were subsequently assigned to receive SC/IV ofatumumab or SC/IV ocrelizumab (7.5 mg/kg; n = 4 per group) on Days 63, 70 and 75. On Day 77, mice received a second SC injection of ^111^In-anti-CD19 (5 µg, 5 MBq) and were imaged by SPECT/CT 72 h later.

The dose of 7.5 mg/kg for both ofatumumab and ocrelizumab was chosen following a pilot study that assessed the efficacy of SC versus IV administration of rituximab in the DTH mouse model of MS.

### Treatment with ofatumumab and ocrelizumab in the DTH-TLS mouse model

huCD20 mice with chronic DTH-TLS lesions (N = 48) were randomly assigned to receive SC/IV ofatumumab or SC/IV ocrelizumab (7.5 mg/kg, n = 10 per group), or vehicle (n = 8). Treatments were administered on Days 63, 70 and 75 post-lesion induction, when no BBB can be observed and meningeal TLS are known to have formed (33). After 18 days of treatment, mice were deeply anaesthetized with pentobarbital, and their blood collected *via* cardiac puncture and transferred to EDTA-coated tubes. Mice were then transcardially perfused with heparinized saline, followed by 4% paraformaldehyde (PFA). Organs were post-fixed overnight in 4% PFA at 4°C and subsequently dehydrated in 30% sucrose. Organs were then embedded in optimal cutting temperature (OCT) compound (CellPath Ltd, UK) and stored at -20°C until further use.

### Immunohistochemistry

Frozen sections were cut at 10 µm using a cryostat (Leica Microsystems, UK). IHC was performed using antibodies to glial fibrillary acidic protein (GFAP, 1:800; Dako, Agilent, UK) for astrocytes, ionized calcium-binding adapter molecule 1 (Iba1, 1:2000; Abcam, UK) for microglia, and myelin basic protein (MBP, 1:1000; Abcam, UK) for myelin. IHC was also performed to stain for markers of B-cells (B220, 1:800; BD, UK) and T-cells (CD3, 1:200; BD Biosciences, UK).

In brief, tissue sections were first rehydrated and washed in PBS. Endogenous peroxidase activity was quenched using methanol with 0.3% H_2_O_2_ for 20 min, and Fc sites blocked using 10% serum for 1 h at room temperature. Tissue slices were then incubated in primary antibodies diluted in 1% serum in PBS overnight at 4°C, and appropriate secondary antibodies were applied for 2 h. After several rounds of washing, tissue sections were incubated in 3,3’-Diaminobenzidine (DAB; Sigma-Aldrich, UK) until a satisfactory level of staining was achieved. Stained sections were counterstained with Cresyl violet and dehydrated through graded alcohols (80%, 90%, 100%), cleared with xylene, mounted with non-aqueous mounting medium, and a coverslip applied.

Two sections with the greatest lesion area from each animal were analyzed by light microscopy (Leitz Dialux 20). In the brains, the area of activated microglia or astrocytes in the DTH lesion was traced using a Camera Lucida. Tracings were scanned, scaled, and the area of activated glia calculated using ImageJ. The total number of T- and B-cells in the ipsilateral hemisphere were counted on two sections per animal and the average was calculated. To analyze the extent of B-cell depletion in the spleens and lymph nodes with IHC, entire follicles and B220+ areas were traced using a Camera Lucida, calculated using ImageJ, and used to determine the ratio of the area taken up by B-cells (B220+) in the follicle. All slides were randomized and blinded until analysis was complete.

### Flow cytometry

Splenic, cervical and inguinal lymph node tissue samples were mechanically dissociated through a 70 µm cell strainer using a sterile syringe plunger, and centrifuged at 450 × g for 5 min at 4°C. Splenic cells were resuspended in 500 µL red blood cell lysing solution, incubated for 1 min at room temperature, and subsequently washed in Dulbecco’s PBS (DPBS). Following incubation of all samples with Fc Block (BD biosciences, UK) for 15 min, tissue samples were washed and incubated on ice with anti-CD19 Alexa Fluor 488 (BioLegend, US) in 100 μL RPMI containing 1% FBS for 30 min. Cells were fixed in 2% PFA on ice for 20 min, washed twice and resuspended in 500 μL PBS. Cell data was collected using a FACSCanto II (BD Biosciences, UK). To obtain accurate cell counts, cells were first gated using the forward versus side scatter to remove debris. The cells were then gated for singlets (FSC-H versus FSC-A) and then by CD19 staining. In total, 10,000 singlet events were collected per sample. Representative gating strategy images for the flow cytometry analysis of CD19+ cells are shown in **Supplementary Figure 1**. All samples were analyzed using FlowJo software (v.10.3.0, Tree Star Inc) to determine the percentage of CD19+ cells in the entire spleen or lymph node sample.

### Statistical analysis

All statistical analyses and non-linear regression were performed using GraphPad Prism version 7 (GraphPad Software, San Diego). Data were tested for normality using a Shapiro-Wilk test. A two-way analysis of variance (ANOVA) was used to calculate the impact of treatment over time. A one-way ANOVA followed by Tukey’s *post hoc* test was used to calculate the significance of difference between groups. All data were obtained at least in triplicate and results reported as mean ± standard error of the mean (SEM), unless otherwise stated. When the *p* value was < 0.05, groups were considered to be significantly different.

## Results

### Route of ofatumumab and ocrelizumab administration alters antibody distribution in huCD20 mice

To investigate whether the route of administration of ofatumumab or ocrelizumab alters antibody distribution, ofatumumab and ocrelizumab were radiolabeled with ^111^In. Radiolabeling yields of >95% were confirmed by iTLC, and identical, single bands (~150 kDa) were observed by SDS-PAGE followed by autoradiography (**Appendix B, Supplementary Figure 2**).

**Figure 1:**
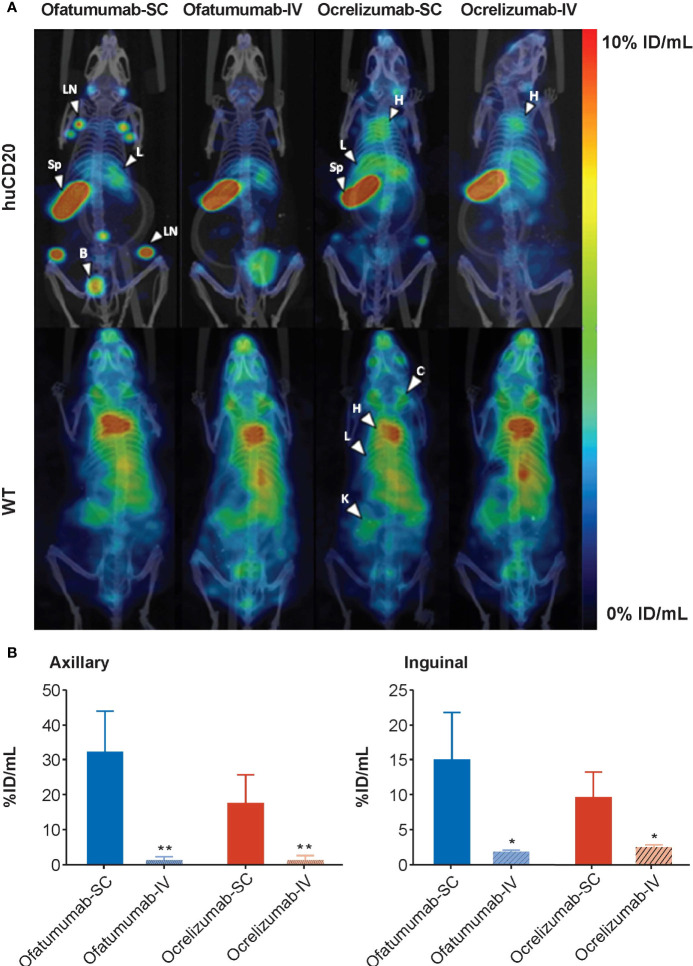
**(A)** Representative maximum intensity projection (MIP) SPECT/CT images of huCD20 (upper panel) and WT (lower panel) mice 72 h after SC or IV administration of ^111^In-ofatumumab or ^111^In-ocrelizumab. Prominent binding of ^111^In-ofatumumab and ^111^In-ocrelizumab is observed in lymph nodes of huCD20 mice following SC administration, but not after IV administration. Both ^111^In-ofatumumab and ^111^In-ocrelizumab displayed similar tissue distributions. No specific binding was observed in WT mice. *B* = bladder, *C* = clavicle, *H* = heart, *K* = kidney, *L* = liver, *LN* = lymph node, *Sp* = spleen **(B)** VOI analysis of SPECT/CT imaging (N = 5 per group). A significantly higher level of binding was observed in axillary and inguinal lymph nodes of huCD20 mice following SC versus IV administration. *p < 0.05; **p < 0.01. Data represent mean ± SEM.

^111^In-ofatumumab or ^111^In-ocrelizumab was subsequently administered SC or IV to huCD20 and WT mice and their distribution assessed by SPECT/CT (**Figure 1A**). The imaging results revealed similar tissue distribution of ^111^In-ofatumumab and ^111^In-ocrelizumab following SC and IV administration; however, an increase in ^111^In-ofatumumab and ^111^In-ocrelizumab was observed in the axillary and inguinal lymph nodes following SC versus IV administration. This improved lymph node targeting with the SC route of administration was confirmed by quantitative VOI analysis (**Figure 1B**). Although the total body retention of ^111^In-ofatumumab appeared stronger than that of ^111^In-ocrelizumab after SC administration, there was no significant difference in axillary and inguinal lymph node uptake between the two antibodies, as determined by VOI image analysis. As expected, no specific uptake of ^111^In-ofatumumab or ^111^In-ocrelizumab was observed in WT mice lacking the human CD20 epitope.

**Figure 2:**
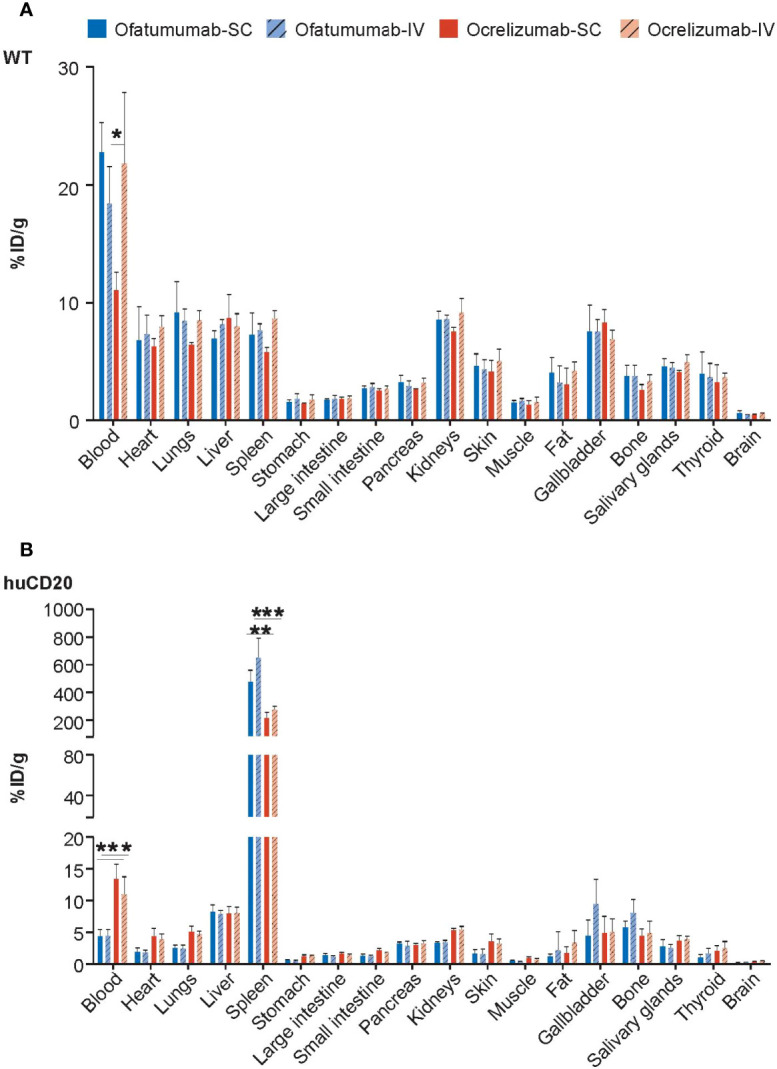
Gamma counting analysis of ^111^In-ofatumumab and ^111^In-ocrelizumab in the blood and individual organs of WT and huCD20 mice. **(A)** Non-specific accumulation of ^111^In-ofatumumab and ^111^In-ocrelizumab was observed in the tissue of WT mice. **(B)** Increased levels of ^111^In-ocrelizumab and ^111^In-ofatumumab were observed in the B-cell rich spleen of huCD20 mice. ^111^In-ofatumumab-treated huCD20 mice displayed faster clearance from the blood and uptake in the spleen following both SC and IV administration compared with ^111^In-ocrelizumab. *p < 0.05; **p < 0.01; ***p < 0.001. Data represent mean ± SEM.

Ex vivo gamma counting analysis was performed to assess the levels of ^111^In-ofatumumab and ^111^In-ocrelizumab in the blood and individual organs of the same huCD20 and WT mice 72 h after administration. As expected, and in agreement with the whole-body imaging studies, neither ^111^In-ofatumumab nor ^111^In-ocrelizumab specifically accumulated in the tissues of WT mice, in which a typical IgG distribution pattern was observed (**Figure 2A**). In contrast, increased levels of ^111^In-ocrelizumab and ^111^In-ofatumumab were measured in B-cell rich compartments (e.g. spleen) of huCD20 mice (**Figure 2B**). Furthermore, ^111^In-ofatumumab-injected mice displayed faster clearance from the blood and higher uptake in the spleen compared with ^111^In-ocrelizumab-injected mice, regardless of the administration route.

### ^111^In-anti-CD19 antibody can detect B-cells in huCD20 and WT mice

**Figure 3:**
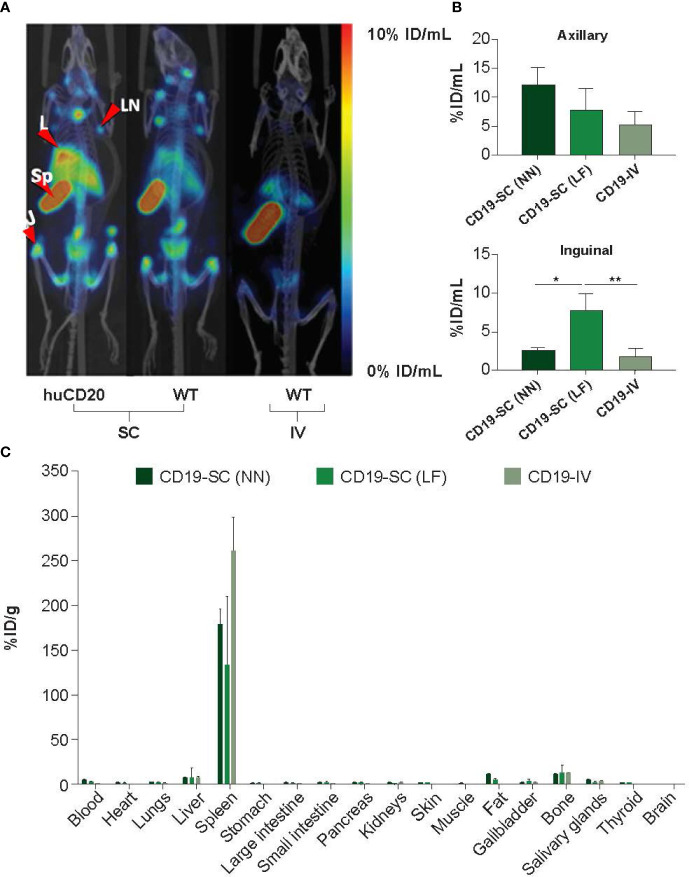
**(A)** Representative MIP SPECT/CT images of huCD20 and WT mice 72 h after SC or IV administration of ^111^In-anti-mouse CD19. Prominent binding of ^111^In-anti-CD19 is observed in lymph nodes of huCD20 and WT mice following SC administration, but not after IV administration. *J* = joint; *L* = liver, *LN* = lymph node, *Sp* = spleen. **(B)** Quantitative VOI analysis of SPECT/CT imaging. **(C)** Uptake of ^111^In-anti-CD19 in the blood and tissue of WT mice 72 h after administration, as assessed by ex-vivo gamma counting analysis. *LF* = right lower flank, *NN* = nape of the neck. *p < 0.05; **p < 0.01. Data represent mean ± SEM.

Anti-CD19 antibody was radiolabeled with ^111^In. Radiolabeling efficiencies of >95% were confirmed by iTLC and single bands were observed by gel electrophoresis (SDS-PAGE) followed by autoradiography (**Appendix B, Supplementary Figure 3**). Both huCD20 and WT mice were administered SC or IV ^111^In-anti-CD19 antibody to determine whether imaging of the CD19 epitope of B-cells could be used as an *in vivo* measure of B-cell depletion. SPECT/CT imaging 72 h later revealed a significantly higher accumulation of ^111^In-anti-CD19 in the spleen and lymph nodes of both huCD20 and WT mice following SC administration compared with IV administration (**Figure 3A**), which was confirmed by quantitative VOI analysis (**Figure 3B**). For this reason, SC administration of ^111^In-anti-CD19 was used to track B-cell depletion in huCD20 mice following SC or IV administration of ofatumumab or ocrelizumab. Ex vivo gamma counting analysis also confirmed the accumulation of ^111^In-anti-CD19 in the B-cell rich spleen (**Figure 3C**).

### Ofatumumab and ocrelizumab significantly reduce ^111^In-anti-CD19 signal in lymph nodes of huCD20 mice with chronic DTH-TLS lesions

The DTH-TLS mouse model was used to assess the impact of ofatumumab and ocrelizumab on B-cell levels in the cervical and axillary lymph nodes. This model features a gradually expanding area of activated microglia and astrocytes, increasingly growing B- and T-cell accumulation, and increasingly worsening demyelination over time (33).

**Figure 4:**
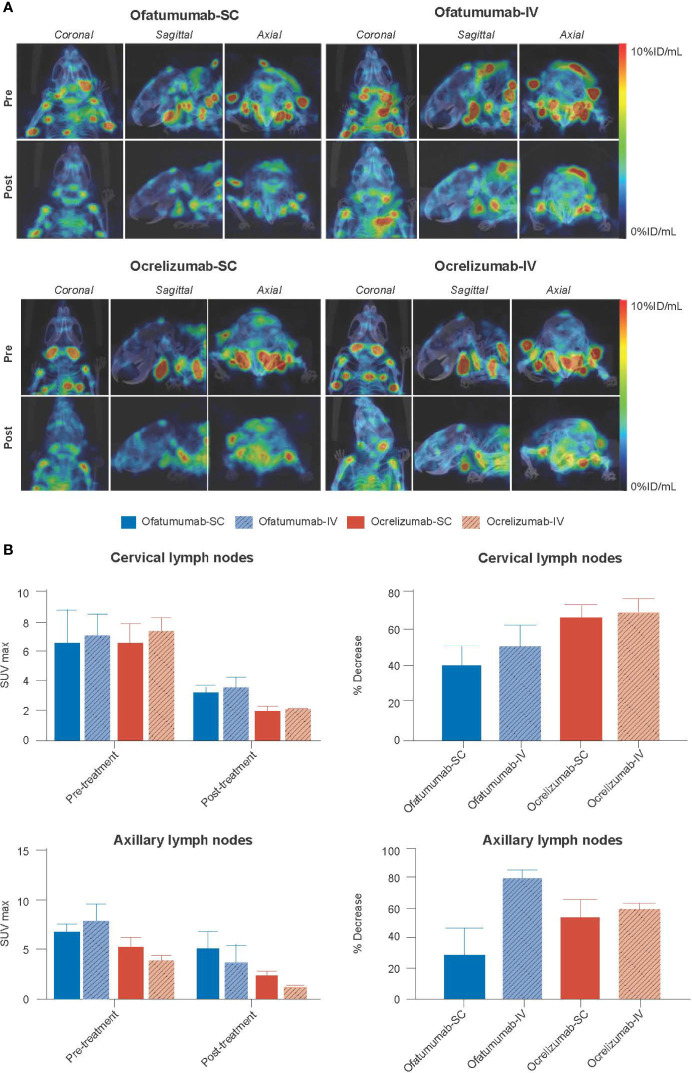
**(A)** Representative MIP SPECT/CT images of huCD20 mice 72 h after SC administration of ^111^In-anti-CD19 (pre) and 72 h after SC administration of ^111^In-anti-CD19 following 18 days of treatment with SC or IV ofatumumab or ocrelizumab (post). A reduction in ^111^In-anti-CD19 signal in lymph nodes, representing B-cell depletion, is noted post treatment. **(B)** Two-way ANOVA revealed that all treatments significantly reduced the ^111^In-anti-CD19 signal over time in the cervical lymph nodes and axillary lymph nodes, but no significant difference in SUVmax was observed between groups (route of administration or antibody). There was also no significant difference in the relative decrease in SUVmax between groups, as assessed by one-way ANOVA. Data represent mean ± SEM.

Since anti-CD20 therapies interfere with detection of the CD20 antigen, CD19+ B-cell levels were used as a proxy to measure the extent of B-cell depletion following anti-CD20 therapy. Mice were first administered SC ^111^In-anti-CD19, 60 days post lesion induction, and baseline SPECT/CT images were captured. This was followed by SC/IV administration of ofatumumab or SC/IV administration of ocrelizumab on Days 63, 70 and 75. Mice were then re-administered SC ^111^In-anti-CD19 on Day 77 and imaged 72 h later by SPECT/CT. The imaging results revealed a significant reduction in ^111^In-anti-CD19 signal in the cervical lymph nodes of huCD20 mice 18 days post treatment initiation with ofatumumab and ocrelizumab (**Figure 4A**). Two-way ANOVA revealed that all treatments significantly reduced the ^111^In-anti-CD19 signal over the treatment period in the cervical lymph nodes (F (1, 16) = 21.87; *p* = 0.0003) and axillary lymph nodes (F (1,16) = 8.19; *p* = 0.0113); however, there was no significant difference between the groups (route of administration or mAb) (**Figure 4B**). There was also no significant difference in the relative decrease in SUVmax between the groups, as assessed by one-way ANOVA.

**Figure 5:**
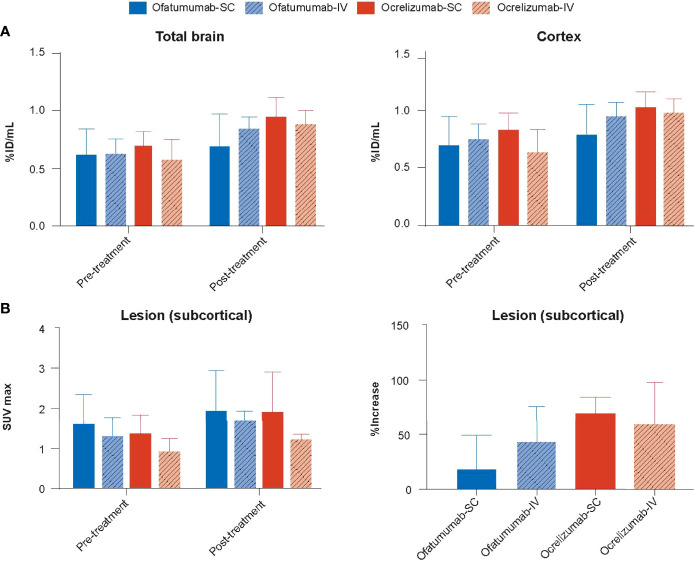
**(A)** The ^111^In-anti-CD19 signal (%ID/mL) was increased in the total brain and cortex following 18 days of treatment with SC or IV ofatumumab or ocrelizumab, as assessed by two-way ANOVA. This observed increase in ^111^In-anti-CD19 signal was lowest in SC ofatumumab-treated mice. **(B)** No significant difference in SUVmax was observed with treatment over time in the lesion, and one-way ANOVA revealed no between group differences. Data represent mean ± SEM.

Very little ^111^In-anti-CD19 signal was detected in the brain of huCD20 mice; however, two-way ANOVA revealed a small but significant increase in signal (%ID/mL) over time in the total brain (*p* = 0.0018) and cortex (*p* = 0.0033) (**Figure 5A**). No significant difference in SUVmax was observed with treatment over time in the lesion, and one-way ANOVA revealed no between group differences (**Figure 5B**).

### Ofatumumab and ocrelizumab significantly reduce the number of CD19+ cells in the spleen and lymph nodes of huCD20 mice with chronic DTH-TLS lesions

**Figure 6:**
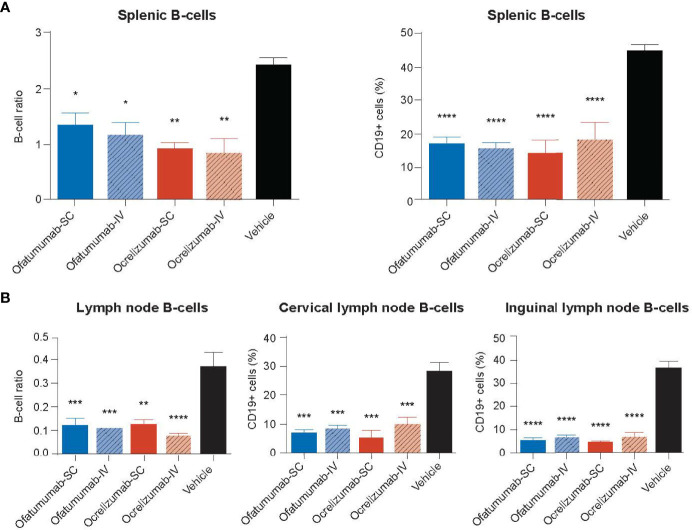
**(A)** The number of CD19+ cells in the spleen and **(B)** cervical and inguinal lymph nodes following treatment with SC or IV ocrelizumab or ofatumumab. IHC and FACS analysis revealed all treatments significantly depleted the number of CD19+ cells in the spleen and cervical and inguinal lymph nodes relative to vehicle after 18 days. *p < 0.05, **p < 0.01, ***p < 0.001, ****p < 0.0001. Data represents mean ± SEM. Vehicle values represent the percentage of the total number of CD19+ cells in the organ.

The DTH-TLS mouse model was also used to assess the impact of SC/IV administration of ofatumumab or SC/IV administration of ocrelizumab on the number of CD19+ cells in the spleen and cervical and inguinal lymph nodes. Treatments were initiated on Days 63, 70 and 75 post lesion induction. Following 18 days of treatment, IHC and FACS analysis revealed that all treatments significantly depleted CD19+ cells in the spleen relative to vehicle (**Figure 6A**). Furthermore, all treatments significantly depleted CD19+ cells in cervical and inguinal lymph nodes relative to vehicle (**Figure 6B**). There was no significant difference between the two antibodies or the route of administration.

### Ofatumumab and ocrelizumab significantly reduce the cellularity of MS-like lesions in the DTH-TLS mouse model of MS

**Figure 7:**
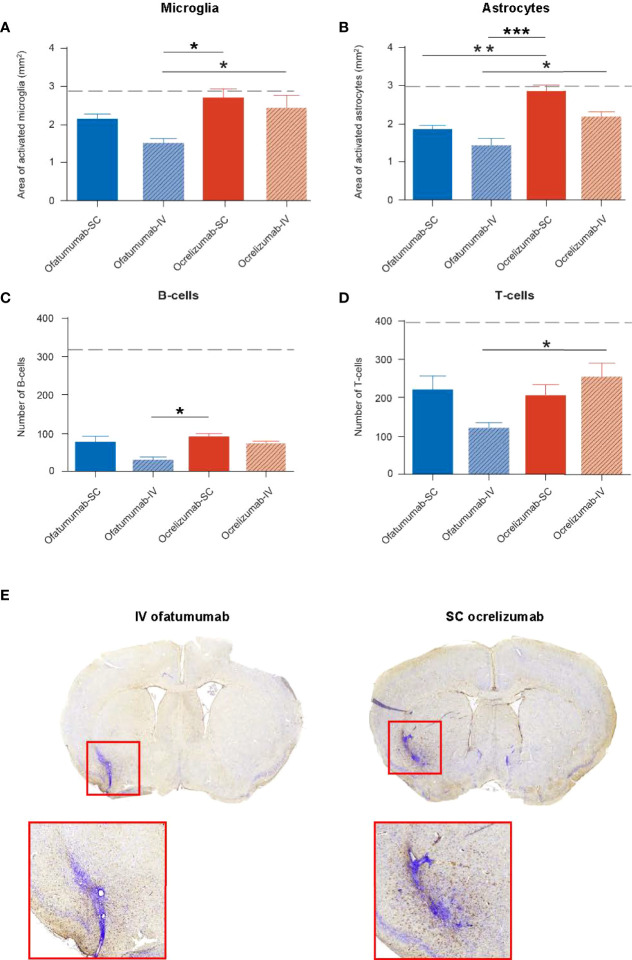
Two-way ANOVA revealed a significant effect of treatment on the area of microglia and astrocyte activation as well as the number of B- and T-cells in the lesion, as assessed by IHC. **(A)** The area of microglia activation was significantly reduced with IV ofatumumab versus IV and SC ocrelizumab. **(B)** The area of astrocyte reactivity was significantly reduced with IV and SC ofatumumab versus SC ocrelizumab, and by IV ofatumumab vs IV ocrelizumab. **(C)** IHC demonstrated a lower number of B-cells in the lesion of mice treated with IV ofatumumab compared with SC ocrelizumab. **(D)** IHC demonstrated a significant reduction in the number of T-cells with IV ofatumumab versus IV ocrelizumab. **(E)** Representative images of microglial activation in the brains of IV ofatumumab-treated mice (left) and SC ocrelizumab-treated mice (right) *p < 0.05, **p < 0.01. Data represents mean ± SEM. Dashed line represents vehicle-treated animals (SEM: microglia, 0.286; astrocytes, 0.4656; B-cells, 86.58; T-cells, 95.9).

The impact of SC/IV administration of ofatumumab or SC/IV administration of ocrelizumab on the evolution of DTH-TLS lesions in the brain was assessed following 18 days of treatment. The model features a focal lesion with surrounding microglia and astrocyte activation, demyelination, and B- and T-lymphocyte infiltration, which were determined by IHC. Two-way ANOVA revealed that for the area of microglial activation (**Figure 7A**), there was a main effect of treatment alone (F (1, 32) = 9.048; *p* = 0.0051) in which ofatumumab was more effective than ocrelizumab. For the area covered by reactive astrocytes (**Figure 7B**), there was a main effect of treatment (F (1, 32) = 20.45; *p* < 0.0001) and route of administration (F (1, 32) = 8.801; *p* = 0.0057), but no interaction. This was also the case for the number of B-cells (**Figure 7C**) present in the lesion (treatment: F (1, 18) = 4.978; *p* = 0.0386; route: F (1, 18) = 7.061, *p* = 0.016). In each instance, ofatumumab was more effective than ocrelizumab and the IV route was more efficacious than the SC route. For T-cells, the interaction was significant (F (1, 18) = 4.416; *p* = 0.05), but there were no main effect of treatment. Tukey *post-hoc* analysis (adjusted p values) revealed that for the area of microglial activation, IV ofatumumab was more effective than either SC ocrelizumab (*p* = 0.012) or IV ocrelizumab (*p* = 0.05). Both SC ofatumumab (*p* = 0.006) and IV ofatumumab (*p* < 0.0001) were more effective than SC ocrelizumab in reducing the area occupied by reactive astrocytes, and IV ofatumumab was more effective than IV ocrelizumab (*p* = 0.04). For B-cells, *post-hoc* analysis showed that IV ofatumumab was more effective than SC ocrelizumab (*p* = 0.02), and for T-cell numbers, IV ofatumumab was more effective than IV ocrelizumab (*p* = 0.045) (**Figure 7D**). Representative images of microglial activation in the brains of IV ofatumumab-treated mice and SC ocrelizumab-treated mice are shown in **Figure 7E**.

Prior to the provision of radiolabeled anti-CD19, we assessed the impact of SC vs IV administration of a generic anti-CD20 mAb (rituximab) on T-cell accumulation, astrocyte reactivity and microglia activation in huCD20 mice with chronic DTH lesions. Findings from this pilot study revealed that the SC route of administration reduced the accumulation of T-cells in the DTH lesions more effectively than the IV route (p < 0.05) (**Appendix B, Supplementary Figure 4**). Astrocyte and microglia activation tended to be lower following SC rituximab versus IV rituximab; however, the difference was not statistically significant.

## Discussion

mAbs that target CD20-expressing B-cells have proven to be an effective therapy for patients with MS (30, 36); however, the functional impact of administration route on biodistribution and MS outcome is not well understood. Furthermore, few studies have directly compared two anti-CD20 mAbs in the same model of MS. In this study, we found similar tissue distribution in huCD20 mice following SC and IV administration of ^111^In-ofatumumab and ^111^In-ocrelizumab, but improved lymph node targeting was observed with SC administration of both antibodies. These experiments also revealed faster clearance from the blood and uptake in the spleen following SC and IV administration of ^111^In-ofatumumab versus ^111^In-ocrelizumab. In addition, the same dose of ofatumumab led to a greater reduction in lymphocyte numbers and glial activation compared with ocrelizumab in the DTH-TLS model of MS.

After decades of B-cells being overlooked in MS disease pathogenesis and progression, we now know that they play an integral role, largely due to the success of B-cell depleting therapies (20–23). The immediate benefit of anti-CD20 agents has been mainly attributed to the depletion of B-cells in the blood and even more so from secondary lymphoid organs, such as lymph nodes and the spleen (37). In this study, ^111^In-ofatumumab and ^111^In-ocrelizumab were both effectively absorbed following SC and IV routes of administration, and while similar tissue distribution patterns were observed, SPECT/CT imaging and quantitative VOI analysis revealed an increase in ^111^In-ofatumumab and ^111^In-ocrelizumab in the axillary and inguinal lymph nodes following SC vs IV administration. In line with previous findings in other experimental models (38, 39), this suggests that there may be more direct access to the lymph nodes through the lymphatic system with SC administration than with IV injection. Interestingly, faster clearance from the blood and uptake in the spleen was observed for ^111^In-ofatumumab versus ^111^In-ocrelizumab, regardless of the route of administration, suggesting ofatumumab may be able to reach its molecular target more efficiently than ocrelizumab, at least in this mouse model.

B-cells express several unique markers on their surface, including CD19 and CD20 molecules, which provide selective targets for mAbs (30). Like CD20, CD19 is found on the surface of pre-, immature, mature, and memory B-cells, but unlike CD20, CD19 can also be found on the surface of pro-B-cells, on the majority of plasma cells in secondary lymphoid organs, and all plasma cells in the blood (40). In this study, CD19 was used as a proxy to measure the extent of B-cell depletion following anti-CD20 therapy with ofatumumab and ocrelizumab. Notably, SPECT/CT revealed a significant accumulation of ^111^In-anti-CD19 in the axillary and inguinal lymph nodes of WT and huCD20 mice following SC vs IV administration, and thus this route of administration was used for later experiments. In the DTH-TLS mouse model of MS, both ofatumumab and ocrelizumab significantly reduced the ^111^In-anti-CD19 signal in cervical and axillary lymph nodes as well as the number of CD19+ cells in the spleen and lymph nodes, thus demonstrating the B-cell-depleting capabilities of these mAbs. Both antibodies at the given dose were equally effective at reducing the ^111^In-anti-CD19 signal, regardless of the administration route. We suspect that the use of 7.5 mg/kg was too high to reveal any advantage for one route of administration over another, as the effect is likely to have plateaued. In other pre-clinical studies, low-dose SC and high-dose IV administration of anti-CD20 therapy resulted in similar depletion of CD20+ B-cells in circulation and in lymph nodes (41–43). Thus, it seems probable that a dose-response study would reveal differences between the route of administration and potentially between the two mAbs that cannot be resolved at the higher concentrations employed here. Very little ^111^In-anti-CD19 signal was detected in the brain of huCD20 mice, which may be due to the sensitivity of the method or possibly due to the limited BBB permeability of intact antibodies; however a small increase in signal was detected with treatment. We suspect this may be attributable to the loss of binding sites in the periphery rather than growth of the lesion. Indeed, IHC analysis revealed that the lesion size was diminished by treatment.

Understanding the role of B-cells in MS pathology and the ability to assess the efficacy of B-cell depleting therapies have been hampered by the lack of MS models that recapitulate the B-cell-associated histopathological features of the disease. We recently developed a new rodent model of progressive MS, in which the focal lesions mimic histological features of the disease, including microglia activation, astrogliosis, demyelination, and B- and T-cell infiltration (33). Importantly, the lesions are clinically silent, which reflects the increased relevance of centrally compartmentalized demyelination/neurodegenerative pathophysiological processes and the reduction in acute inflammatory/relapse activity that is typical of the progressive phase of the disease (44), and further allows for longitudinal studies and assessment of treatment paradigms. Furthermore, the model features structured and long-term aggregates of B-cells in the meningeal-brain parenchymal interface, representing TLS. It should be noted that at Day 60 there is no remaining BBB breakdown in this model, and the lesions continue to grow and evolve behind an intact BBB as they do in individuals with MS. While such chronic active/slowly expanding lesions are mostly described in progressive MS (45–47), they are also present in patients with RMS (48, 49), and have been linked with disability accumulation (50). Treatments that impact the evolution of chronic active lesions may therefore help delay disability accrual, particularly when administered in the earliest stages of disease. Using this model, we assessed the impact of SC vs IV ofatumumab and ocrelizumab on the progression of this MS-like lesion. Both ofatumumab and ocrelizumab significantly decreased the extent of microglia activation and astrocyte activation, as well as the number of B- and T-cells in the lesion following SC and IV administration. Moreover, at the same dose, ofatumumab was more effective than ocrelizumab at controlling MS-like pathology in the brain. In line with this, preliminary data in patients with relapsing MS suggest that ofatumumab treatment may be associated with decreased microglial activation in cortical grey matter (51). Such findings may prove crucial given the role of activated microglia in the demyelination and neurodegeneration process of MS (52). We have previously shown that anti-CD20 therapy can reduce the number of myelin-phagocytosing macrophages in this model (33); however, it remains to be established if pharmacological reduction of microglial activation translates into improved myelin repair and outcomes. Our findings in the DTH-TLS model suggest that the IV route of administration lent the best protection by ofatumumab, though this could be a reflection of the high dose used in this study. In contrast, findings from our pilot study assessing the benefit of SC vs IV rituximab in the DTH model of MS, revealed that B-cell depletion by SC rituximab was more effective than IV rituximab.

There are, of course, limitations when assessing human mAbs in mouse models of disease in terms of translational relevance and interpretation. For example, while ofatumumab and ocrelizumab elicit similar ADCC activity, ofatumumab primarily induces B-cell depletion *via* CDC in patients with MS (30, 53); however, mouse serum has low intrinsic complement activity and contains complement inhibitors, suggesting a different mode of action may be triggered. That being said, it was recently demonstrated that bone marrow-liver-thymus humanized mice could recapitulate the CDC activity of ofatumumab, with the pharmacokinetics mimicking those observed in humans (54); thus, we know that in this model, at least, the CDC effector function of ofatumumab is not affected. It should also be noted that ofatumumab is a fully human anti-CD20 mAb while ocrelizumab is a humanized mAb; therefore, the immune reactions mounted by the huCD20 mice in this study likely differ due to the possibility of developing anti-human immune reactions. As a result, these findings may not fully translate to humans, especially with regard to the kinetic/distribution profiles of ofatumumab and ocrelizumab after chronic use. Furthermore, the dose of 7.5 mg/kg is substantially higher than that used for SC administration of ofatumumab in clinical practice (20 mg once monthly), which may also limit its translational relevance. Future studies should consider assessing drug doses that are more equivalent to human doses. Finally, while the mouse model employed in this study recapitulates the principal B-cell associated histopathologic features of the disease, enabling better assessment of B-cell targeted therapies, no model can reproduce the totality of MS pathology. For example, in this study, we selected a model where the target CNS antigen, MOG, is known to be central to MOG-antibody disease, but it’s role in MS is unclear.

In conclusion, our findings suggest that SC delivery of anti-CD20 therapies leads to improved lymph node targeting compared with IV administration, which may in turn lead to improved efficacy given these are the sites where pathogenic B- and T-cells interact (19, 55). While both ofatumumab and ocrelizumab were effective at depleting B-cells, ofatumumab was more rapidly cleared from the blood and taken up by the spleen than ocrelizumab and was more effective at reducing the inflammatory response in the brain. Future studies will aim to determine whether the same outcomes can be achieved at lower and more clinically relevant doses of ofatumumab.

## Data availability statement

The raw data supporting the conclusions of this article will be made available by the authors, without undue reservation.

## Ethics statement

The animal study was reviewed and approved by UK Home Office under license P996B4A4E.

## Author contributions

Conception and design: GW, DCA. Synthesis and analysis of labelled antibodies: GW, RK, JBT, JR. Acquisition of data: JBT, JR, MS. Analysis and interpretation of data: MZ, MB, JBT, JR, RK, DL, BC, DCA. Drafting the manuscript and revising for intellectual content: All. Final approval of completed manuscript: All

## Funding

This study was funded by Novartis Pharma AG, Basel Switzerland.

## Acknowledgments

We would like to thank Gaelle Elain for providing the anti-CD19 antibody validation data and Janis Noonan for providing medical writing support, which was funded by Novartis.

## Conflict of interest

MZ, MB, RK, and GW report employment by Novartis. DL is a former employee of Novartis.

The remaining author declares that the research was conducted in the absence of any commercial or financial relationships that could be construed as a potential conflict of interest.

## Publisher’s note

All claims expressed in this article are solely those of the authors and do not necessarily represent those of their affiliated organizations, or those of the publisher, the editors and the reviewers. Any product that may be evaluated in this article, or claim that may be made by its manufacturer, is not guaranteed or endorsed by the publisher.
