# Supplementary material for: Distribution and efficacy of ofatumumab and ocrelizumab in humanized CD20 mice following subcutaneous or intravenous administration
Source: Front Immunol. 2022 Jul 28;13:814064. doi: 10.3389/fimmu.2022.814064 (PMC9366925; doi:10.3389/fimmu.2022.814064)
Supplement: Supplementary file 1 [file DataSheet_1.pdf]

Supplementary Material

1 Appendix A

1.1 Assessment of DTPA-conjugated anti-mouse CD19 binding properties by flow cytometry

CD19+ mouse B-cells (line 300.19) were used in this assay. Approximately, 100,000 cells/well were incubated in the presence of a clathrin inhibitor (Pitstop; Sigma-Aldrich, UK) at a concentration of 25 µM in DPBS for 15 min on ice. DPTA-conjugated or unconjugated anti-mouse CD19 were then added to the cells in DPBS containing 1% fetal calf serum and 5 mM EDTA (Sigma-Aldrich, UK). After 30 min on ice, cells were washed and incubated with anti-mouse IgG (H+L) Alexa 633 (Thermo Fisher Scientific, Massachusetts, USA) for a further 30 min on ice. Cells were then washed and fixed in 2% PFA for 20 min on ice, followed by two more washes. Samples were acquired on a BD™ LSR II (BD Biosciences, UK) and data were analyzed using FlowJo™ software. Antibody titration curves were analyzed using GraphPad Prism with non-liner regression (variable slope, 4 parameters). Based on three independent experiments, the EC_{50} was determined to be 0.83 ± 0.04 nM for the DTPA-conjugated anti-mouse CD19 antibody and 0.64 ± 0.08 nM for the unconjugated antibody.

1.2 SPECT/CT

For the distribution studies, SPECT data was acquired (1 frame, 10 min per frame, 16 positions, 37.5 s per bed position using list mode acquisition) using an ultra-high resolution rat/mouse 1.8 mm collimator, followed by a cone-beam CT scan (55 kV, 0.19 mA, 20 ms) for anatomical reference and attenuation correction.

For treatment effect studies, SPECT/CT data was acquired using the following image acquisition parameters: 1 frame, 10 min/frame, 4 positions, 2.5 min/position, 1.8 mm collimator; ultrafocus CT, normal mode.

SPECT images were reconstructed using U-SPECT-Rec3.22 software (MILabs, Utrecht, Netherlands), applying a pixel-based algorithm with 8 subsets, 6 iterations and 0.8 mm^3 voxel size for ^{111}In (energy window of 159-278 keV, background weight 2.5). To allow quantification of SPECT data, calibration factors derived from ^{111}In phantoms were used. SPECT images were registered to their corresponding CT and attenuation corrected. Quantification of SPECT images using VOI analyses was performed using the PMod software package (Version 3.807, PMOD Technologies), to calculate the percentage of the injected dose (ID) per milliliter per VOI (%ID/mL) per time bin. For the analysis of mouse brain data, the VOI template Mouse_M.Mirrione was used. The average of the three highest concentrations of activity per voxel in a spherical VOI drawn within axillary or cervical lymph nodes was divided by the ID normalized by the weight of the mouse to give maximum standardized uptake values.

2 Appendix B

2.1 Supplementary Figure 1

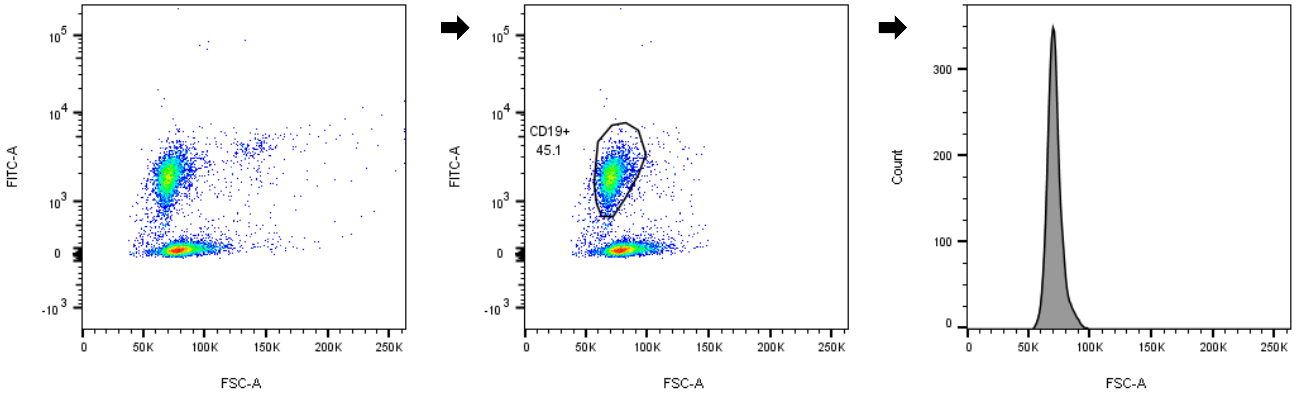

Representative gating strategy for the FC analysis of CD19+ cells in the spleen, inguinal and cervical lymph nodes. Cell data was collected using a FACSCanto II (BD Biosciences, UK). To obtain accurate cell counts, cells were first gated using the forward versus side scatter to remove debris. The cells were then gated for singlets (FSC-H versus FSC-A) and then by CD19 staining of the appropriate cell marker. In total, 10,000 singlet events were collected per sample. All samples were analyzed using FlowJo software (v.10.3.0, Tree Star Inc) to determine the percentage of CD19+ cells in the entire spleen or lymph node sample.

2.2 Supplementary Figure 2

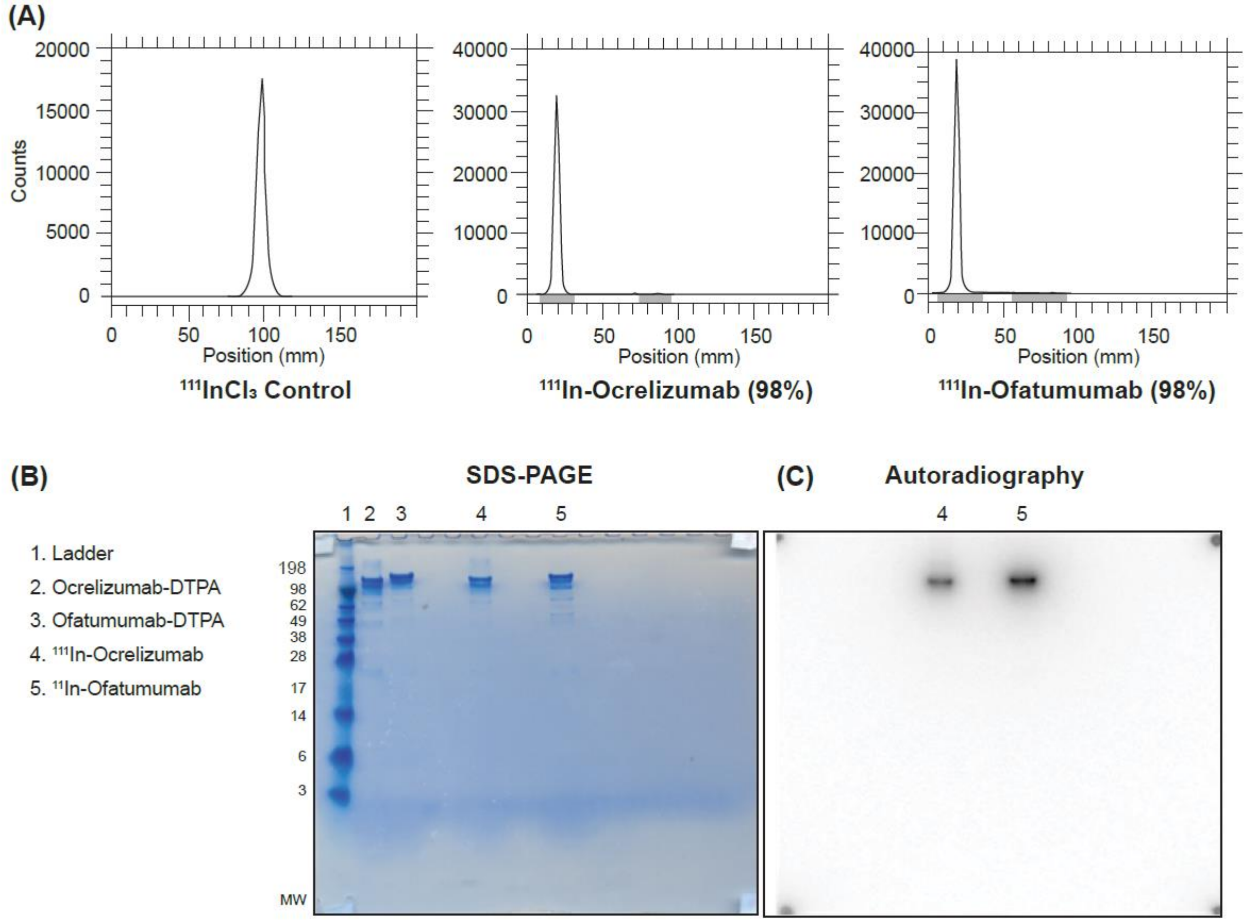

(A) Radiolabeling efficiencies of >95% were confirmed by iTLC, and final antibody concentration was measured by Nanodrop spectrophotometry. (B) Single bands for ^{111}In-ocrelizumab and ^{111}In-ofatumumab, as shown by SDS-Page and (C) by autoradiography.

2.3 Supplementary Figure 3

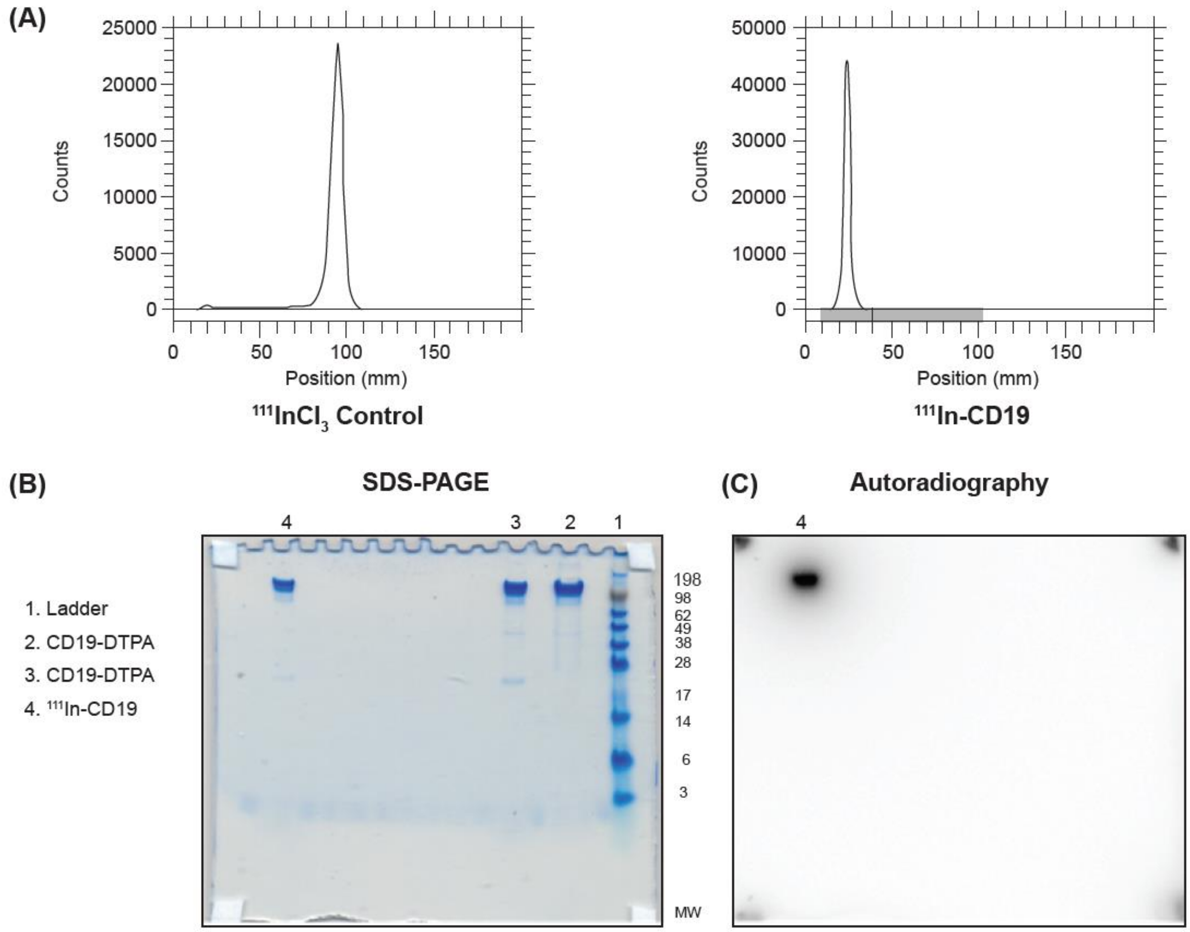

(A) Radiolabeling efficiencies of >95% were confirmed by iTLC, and final antibody concentration was measured by Nanodrop spectrophotometry. (B) Singles bands for ^{111}In-anti-CD19, as shown by SDS-Page and (C) by autoradiography.

2.4 Supplementary Figure 4

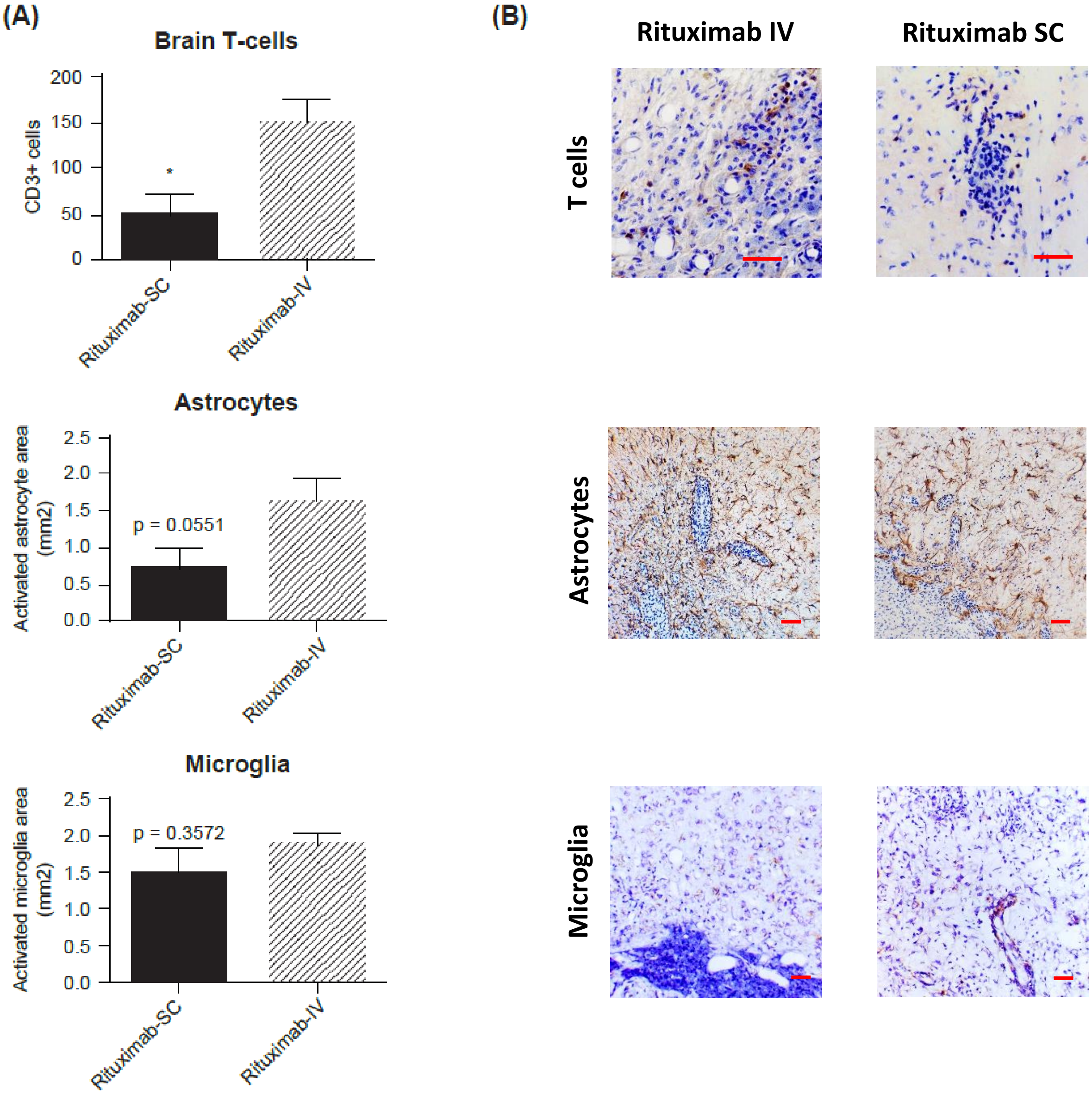

(A) SC rituximab reduced the accumulation of T-cells in DTH lesions more effectively than IV rituximab. Astrocyte and microglia activation tended to be lower with SC administration compared with IV administration of rituximab, but the difference was not statistically significant.
(B) Representative IHC staining of astrocyte activation around the brain lesion of huCD20 mice with DTH lesions. *$p < 0.05$. Data represents mean- ± SEM. Scale bar = 50 µM.
